# The effect of a multi-disciplinary obesity intervention compared to usual practice in those ready to make lifestyle changes: design and rationale of Whanau Pakari

**DOI:** 10.1186/s40608-015-0068-y

**Published:** 2015-10-08

**Authors:** Yvonne C. Anderson, Lisa E. Wynter, Kris R. Moller, Tami L. Cave, Gerard M. S. Dolan, Cameron C. Grant, Joanna M. Stewart, Wayne S. Cutfield, Paul L. Hofman

**Affiliations:** Taranaki District Health Board, New Plymouth, New Zealand; The Liggins Institute, The University of Auckland, Auckland, New Zealand; Sport Taranaki, New Plymouth, New Zealand; Department of Paediatrics, The University of Auckland, Auckland, New Zealand; Centre for Longitudinal Research – He Ara ki Mua, The University of Auckland, Auckland, New Zealand; Starship Children’s Hospital, Auckland District Health Board, Auckland, New Zealand; Department of Epidemiology and Biostatistics, The University of Auckland, Auckland, New Zealand

**Keywords:** Pediatric, Obesity, Research methods, Indigenous people, Whanau Pakari, Intervention studies, Randomised controlled trials, Nutrition, Physical activity, Body mass index

## Abstract

**Background:**

Child obesity internationally has been identified as one of the major threats to future population health. Indigenous people and those from lower socio-economic backgrounds are over-represented in obesity statistics. There is a need for evidence of effect of interventions for child obesity with long-term follow-up. Whether engaging with those that are more motivated to make lifestyle changes is a useful strategy has not been fully explored. We hypothesise that in obese/overweight children, assessed as psychologically “ready for change”, delivery of a 12-month multi-disciplinary intervention programme results in a significant reduction in body mass index standard deviation score.

**Methods/Design:**

Whanau Pakari is an unblinded randomised controlled clinical trial comparing a 12 month intervention programme with standard practice, with 6 monthly assessments for 2 years, conducted in Taranaki, New Zealand (a region where 15.8 % of the population are indigenous). It specifically targets indigenous people and those in more deprived households.

Obese/overweight children and adolescents aged 5–16 years are eligible. Exclusion criteria are medical/psychological conditions leading to inability to undertake physical activity/participate in group sessions; those not “ready” to make lifestyle changes; and those without a committed family member.

Assessments of health parameters, dietary history, physical activity and overall health-related quality of life/psychological functioning are completed in the participant’s home. Fasting blood tests are obtained at baseline, 12 and 24 months.

The primary outcome is body mass index standard deviation score. Secondary outcomes include quality of life, dietary behaviour and physical activity, cardiovascular and metabolic profile (blood pressure, resting heart rate, waist circumference), glycaemic control (fasting glucose and glycated Haemoglobin), fasting insulin, and lipids.

A general linear mixed model will be used to assess change from baseline using the 6, 12, 18 and 24 month measures, adjusting for age, gender, socioeconomic status and ethnicity, and whether at the contemplative or preparation/action stages of readiness for change.

**Discussion:**

This trial will inform the development of management programmes for obese children and adolescents that are appropriate for indigenous populations. It will investigate whether those at the preparation/action stage of “readiness” to make lifestyle changes are more successful in making changes than those who are contemplative.

**Trial registration:**

Australian New Zealand Clinical Trials Registry (ANZCTR):12611000862943. (Date registered 15/08/2011).

## Background

Childhood obesity causes substantial morbidity, mortality and health cost [1, 2]. The rapid increases in the proportion of the population that are overweight and obese are now apparent in children as well as adults in both developed and developing countries [1, 3]. New Zealand has not avoided the global obesity epidemic, with the country now being ranked fourth in the Organisation for Economic Cooperation and Development (OECD) rankings for overweight and obesity (65 % of the population over 15 years are classed as either overweight or obese) [4]. The Health of New Zealand Children Survey 2013/2014 reported 10 % of children aged 5–14 years are obese, up from 8 % in 2006/2007 [5]. Rates of obesity are higher for Maori – New Zealand’s Indigenous population (15 %), Pacific Island children (25 %), and children living in the most deprived quintile of households (18 %) [5].

Few children and adolescents struggling with weight issues have access to intervention programmes. In New Zealand, most overweight or obese children and adolescents coming to the attention of medical professionals are either managed by a general practitioner or general paediatrician with minimal intervention programmes being available nationally [6]. No national cohesive approach for managing childhood obesity exists, despite national clinical guidelines being available since 2009 [7]. A recent multi-centre audit showed that, irrespective of type of intervention, a small but significant reduction in BMI SDS was achievable (−0.15 overall), highlighting the importance of health professionals being proactive in identifying and addressing child obesity [8]. However, even when obesity is identified, intervention is infrequently implemented. In Australia, general practitioners were recently shown to provide weight management for <2 % of overweight and obese children attending primary care services [9], and paediatricians reported lacking confidence in the management of obesity in children, with only 37 % reporting training in the management of obesity-related comorbidities [10]. This is also likely to be the situation in New Zealand, although to date, similar surveys remain unpublished.

Past meta-analyses have supported multi-disciplinary intervention programmes for addressing child and adolescent obesity, as they are deemed as having the greatest chance of success [11, 12]. New Zealand’s Ministry of Health guidelines support an approach of working with family to address food habits, increase physical activity and to promote behavioural change [7]. Evolution of the current trial came from these recommendations, findings from an audit of an existing physical activity/nutrition programme [6] and recognition of the need to address accessibility for those most affected by obesity. A trial was deemed necessary to ensure future decision-making with regard to child and adolescent obesity was informed by the most reliable evidence possible. The trial has been designed with consideration of the Consolidated Standards of Reporting Trials (CONSORT) 2010 statement [13].

It was clear in the pre-existing regional programme that the lack of a measure for assessing a participant’s psychological “readiness” to make lifestyle changes was affecting interpretation of the outcomes of the programme overall [6]. Whilst most clinical practitioners assess readiness to make lifestyle changes in their patients on a daily basis, this is a poorly defined process. Historically, readiness for change (RFC) has been utilised qualitatively in some obesity services. It is a concept that has developed from the transtheoretical model defining stages of behavioural change around addiction [14]. When deciding to undertake behavioural change, an individual moves through defined stages at different rates and not always in a linear fashion (Table 1). However, an individual’s readiness to change may be behaviour-specific, so it is unclear whether a participant’s “readiness” may equate to global changes related to improving lifestyle. In the original readiness for change questionnaire directed towards excessive alcohol use, this was somewhat mitigated by the use of multiple descriptive statements across pre-contemplation, contemplation and action [15].

**Table 1:** Theoretical model of stages of readiness for change [14]

Stage	Description
Pre-contemplation	“I do not have a problem”
Contemplation	“I may have a problem”
Preparation	“I may have a problem and need to do something”
Action	“I will try these changes”
Maintenance	“The changes I have made are now part of what I do”

The transtheoretical model has been used to assess individual’s motivation for smoking cessation [16], and various tools for readiness for change have been trialled in the obesity setting [17, 18]. The utility of readiness for change remains unclear as it relates to obesity services, and it is too simplistic to expect that every individual would move through these stages in a similar fashion. However, previous studies have highlighted the importance of tailoring interventions to the individual stage of change rather than treating all participants as if they are in preparation or action stages [17]. If there were a quantitative tool that could predict likelihood of success at initial assessment, this would potentially focus health resource where it was most likely to make a difference. Given the complexity of behaviour change as it relates to obesity, the original readiness for change questionnaire [15] would require modification and expansion to include questions regarding eating behaviour, attitude towards weight and physical activity behaviour. Confidence to make changes in physical activity and eating behaviour would also need to be considered.

The question remains as to whether an individual’s readiness in the “snapshot” situation of an assessment translates to ongoing motivation to make lifestyle changes over time. In the domain of child and adolescent obesity, parental readiness is a vital factor in a child or adolescent’s success in making and maintaining lifestyle changes. If a parent believes their child’s weight is a problem, or that they as parents are overweight, they are more likely to be ready to make lifestyle changes for their child [18].

“Whanau Pakari” means “healthy self-assured families that are fully active” in Maori, and was the name gifted to the trial by a prominent Maori community representative. The trial assesses a new mainstream clinical service delivered in an innovative way in order to improve access for Maori, and those from lower socioeconomic backgrounds. Previous child obesity services in the region involved the traditional model of medical referral to a Paediatrician, sometimes with the addition of dietitian input, and physical activity programme input. The physical activity programme, run in the community through the regional sports trust with a maximum number of participants per year, did not specifically target high-risk groups, and was not region-wide [6]. The new programme removes the “hospital visit”, but participants continue to receive support and oversight from a Paediatrician. This multi-disciplinary programme incorporates three novel approaches:

1. It provides a trained roving community coordinator to meet families in their homes, enabling improved access to the clinical service, especially for Maori.
2. It assesses “readiness for change” both quantitatively and qualitatively.
3. It provides follow up to 12 months post intervention to assess persistence of lifestyle changes, which is longer than any other multi-disciplinary obesity intervention model trialled in New Zealand.

There is a clear need for evidence-based interventions for child obesity that demonstrate on-going effectiveness [11]. Whether engaging with those that are more motivated to make lifestyle changes is a strategy that warrants further exploration. Our objectives are firstly, to undertake a multi-disciplinary intervention, which is accessible and appropriate for those most affected by child obesity. Secondly, we aim to assess whether a quantitative RFC questionnaire is useful in predicting response to the intervention. We hypothesise that in obese/overweight children, assessed as psychologically “ready for change”, delivery of a 12-month multi-disciplinary intervention programme results in a significant reduction in body mass index standard deviation score at 12 months.

## Methods/Design

Whanau Pakari is an unblinded randomised controlled clinical trial being conducted in Taranaki, New Zealand (population 23,139 children aged 0–15 years, of which 28 % are Maori) [19]. Ethical approval was granted by the Health and Disability Ethics Committee (Ministry of Health, New Zealand; CEN/11/09/054), and the trial was registered with the Australian New Zealand Clinical Trials Registry (ANZCTR: 12611000862943). Locality approval has been obtained from Sport Taranaki, and the Taranaki District Health Board.

### Participants

Children from the Taranaki region aged 5–16 years, with a body mass index (BMI) ≥98th centile, or those >91st centile with weight related comorbidities will be offered participation in the trial if referred to “Whanau Pakari”. These cut-offs are a modification of UK Cole data, and have been chosen as they are nationally accepted for use by the Ministry of Health for defining obesity and overweight respectively in the community for 0–5 years [20, 21]. (There are no nationally accepted growth charts or official cut-offs in use for >5 year old children currently.)

Exclusion criteria will be significant medical or psychological conditions leading to inability to undertake physical activity or participate in group sessions; those not “ready” to make lifestyle changes; and those without a committed family member (essential to support the family-based approach of the programme).

**Fig. 1:**
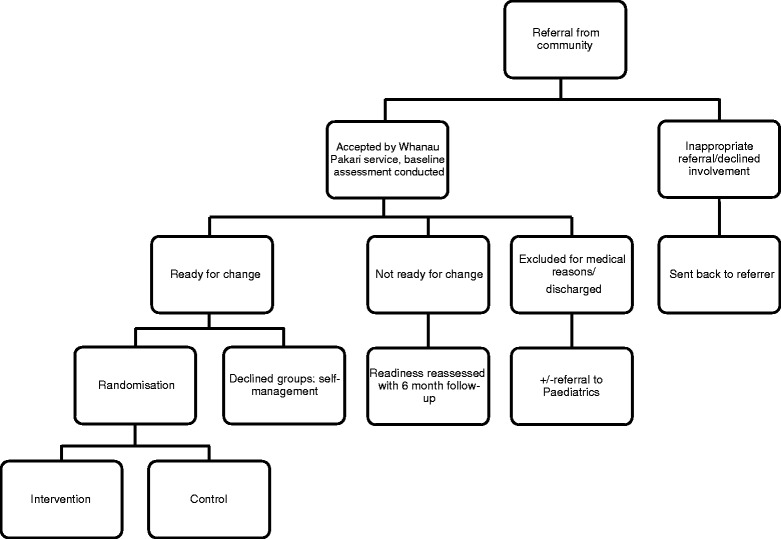
Summary of Whanau Pakari trial design within service

To optimise accessibility, referrals will be accepted from all health professionals within the community, including public health nurses in schools, and Maori health workers. Self-referrals will also be accepted (Fig. 1).

### Recruitment

Recruitment for the trial will be through the service, which is being advertised across Taranaki through multiple channels, including referrer training half-days (training sessions about the service, and how to have conversations about weight with families), meetings (hui) with all stakeholders, general practice visits, school visits, media releases, pamphlet drops at public places and events, and the Public Health Unit of Taranaki District Health Board.

Whilst the trial will assess a “mainstream” clinical service, the aim is to target Maori and the most deprived in the community. To achieve this aim we will use identified facilitators that enable Indigenous people’s participation such as relationship and partnership building, involvement of Indigenous staff, the use of Indigenous knowledge models (such as He Korowai Oranga: the Maori Health strategy and the Whanau Ora [healthy families] tool) [22, 23], targeted recruitment techniques (such as working with Maori health workers within the community to engage with families) and adapting study material [24].

All children referred will be seen in the family (whanau) home by the Healthy Lifestyles Coordinator (a health professional trained in focussed weight-related assessment, supported by a Paediatrician). This design aspect allows the trial to run as a community-based service yet with a full clinical component to it. Removal of a hospital-based appointment with a specialist was designed to enable access to the service for larger numbers and minority groups, and to avoid “medicalisation” of the process. Given the service is targeting Maori and those from lower socio-economic areas, ensuring accessibility and appropriateness are crucial to the study design, without compromising clinical care.

### Conception and consultation

The multi-disciplinary intervention model was developed after consideration of the national guidelines for implementation of weight management programmes, and the recommendations from the international systematic reviews and meta-analyses [7, 11, 12]. Longer-term follow-up was deemed imperative to determine whether lifestyle changes persisted over time, which has been a deficit of many previous studies. Conceptually, a multi-disciplinary intervention that incorporated dietary, physical and psychological support was considered important, with 12 months the optimal length of intervention. It was necessary for the intervention to be community-based with a strong focus on accessibility to Maori, and those from lower socio-economic areas.

Guidance was sought in the inception stages from the Maori Health Unit of Taranaki District Health Board, who advised appropriate linkages with the community, identifying the key Maori stakeholders from Maori Health Centres in the region, and tribal representatives. Maori were instrumental in the initial set-up phase, applying a Maori lens to service delivery and curriculum development, and were on the interview panel for all staff members. It was imperative to the researchers that the consultation undertaken helped to:

1. Build appropriate and positive relationships around the region, particularly in South Taranaki, which has a proportionally larger population of Maori.
2. Ensure acceptability of the intended research by using a community-based approach.
3. Provide an opportunity for input and contribution from community stakeholders during the consultation process.

This process ensured that potential barriers for engagement for Maori and the most deprived in the community such as lack of access, unfamiliarity with research, distrust and problems with the research material were addressed [24]. The consultation process is ongoing.

### Assessments

Assessments will be completed at six monthly intervals for 2 years from enrolment. Information will be gathered regarding ethnicity, past medical history, medical conditions and family history. Written informed consent will be obtained to take part in the trial and to share collected information between Sport Taranaki (the regional sports trust) and the Health Board. This will be from the participant if age-appropriate, or where the participants are children, a parent/guardian. Table 2 shows the key elements of the assessments undertaken.

**Table 2:** Assessment information for all participants

Key assessments	Baseline	6 months	12 months	18 months	24 months
Resting heart rate	✓	✓	✓	✓	✓
Blood pressure^a^	✓	✓	✓	✓	✓
Height^b^	✓	✓	✓	✓	✓
Weight^c^	✓	✓	✓	✓	✓
Waist circumference^d^	✓	✓	✓	✓	✓
Hip circumference^e^	✓	✓	✓	✓	✓
Peak flow^f^	✓	✓	✓	✓	✓
Acanthosis nigricans screen	✓	✓	✓	✓	✓
Ear, nose and throat examination^g^	✓	✓	✓	✓	✓
Self report of Tanner pubertal stage^h^	✓	✓	✓	✓	✓
Accompanying adult’s height and weight	✓		✓		✓
Questionnaires^i^	✓	✓	✓	✓	✓
Blood sampling^j^	✓		✓		✓

Technical/procedural information: ^a^using Welch Allyn portable sphygmomanometer with flexiport reusable blood pressure cuffs of appropriate size, ^b^to 0.1 cm using average of three readings on Seca 213 portable stadiometer, ^c^to 0.1 kg using Seca 813 digital scales, ^d^Seca 201 standard measuring tape (at mid-point between the lower margins of the rib and the top of the iliac crest to 0.1 cm at end of normal expiration) [52]), ^e^widest girth, ^f^using Mini Wright peak flow meter, ^g^using Welch Allyn portable auroscope, ^h^or from parent in very young children [31], ^i^apart from RFC questionnaire (only performed at baseline), ^j^fasting insulin, fasting glucose, liver function tests, C-reactive protein, glycated Haemoglobin (HbA1c), and fasting lipids

#### Calculations

BMI, BMI percentile and BMI standard deviation score (SDS) will be calculated using UK Cole normative data [25] on the uploadable KIGS auxology software (Pfizer Endocrine Care TM). Height percentile will be calculated using gender specific growth charts for 2–18 years recommended by the Australasian Paediatric Endocrine Group for Australian and New Zealand use [26], based on Centers for Disease Control stature for age and weight for age data [27]. To improve accuracy, BP SDS will be calculated using an age-based paediatric blood pressure reference chart calculator based on data from The Fourth Report [28, 29]. BP SDS will then be converted to percentiles. Peak flow percentile will be calculated based on reference New Zealand peak expiratory flow rates [30]. Waist hip ratio, and waist height ratio (WHtR) will be calculated. Participants will be deemed pubertal if they are female with breast development ≥ B2 or male with pubic hair development ≥ P3 on Tanner pubertal staging [31]. Level of household deprivation will be calculated based on the New Zealand Deprivation Index 2006 [32].

#### Questionnaires

One aim of this trial is to determine whether there is an association between reported degree of “readiness” to make lifestyle changes at baseline assessment, and improvements in lifestyle, including BMI SDS. Readiness for lifestyle change will be based on the transtheoretical model of stages of change [16], and will be established with two questionnaires (parent and child/adolescent) and the best judgement of the Healthy Lifestyles Coordinator at the end of the baseline assessments. Both questionnaires will be based on the 12-item Readiness to Change Questionnaire [15] and will use a 5-point Likert scale to assess the parent’s and child/adolescent’s beliefs, attitudes and behaviour about weight, eating behaviour and physical activity. Given the complexity of obesity, the questionnaires will be expanded. The child questionnaire will have 21-items and will be used for children 11 years and older, and a 27-item parent questionnaire, including 6 questions regarding family attitudes/behaviour, will be administered to parents. Whilst self-efficacy/confidence to make changes are not extensively measured, confidence in making changes in physical activity and eating behaviour has been included in both quantitative questionnaires.

Qualitative assessments of readiness for change will be made by the Healthy Lifestyles Coordinator at the end of the assessment. This will be the Coordinator’s overall subjective opinion as a health professional of stage of change in both the committed family member and child (if >11 years of age), based on the assessment. A qualitative stage of change will be included as this is what is undertaken in current clinical practice, and provides a comparator for the quantitative tool. The quantitative assessment will be scored at point of data entry by the Healthy Lifestyles Coordinator. Cronbach’s alpha will be used to establish the reliability of our readiness for change lifestyle questionnaire. Confirmatory factor analysis will be used to examine convergent and discriminant validity. The questionnaire was tested for understanding and comprehension in a randomly selected cohort of clinic patients prior to trial commencement.

Questionnaires that will be administered to the child/adolescent or parent include the Paediatric Quality of Life Inventory (PedsQL)™ – a measurement model designed to evaluate health-related quality of life in children and adolescents that has been extensively validated [33–38], the Achenbach Child Behavior Checklist (CBCL) (Child Behavior Checklist for Ages 1½-5: 7–28-00 Edition-601, Child Behavior Checklist for Ages 6–18: 6-1-01, Edition-201, Youth Self-report for Ages 11–18: 6-1-01 Edition-501) [39], children’s physical activity questionnaire (C-PAQ) [40], modified children’s dietary questionnaire for New Zealand use (CDQ) [41], 24 h food recall, knowledge of healthy lifestyles questionnaire (modified from the 2008 Nutrition Survey) [42], and our RFC questionnaire. Any participant deemed not ready for change will be reassessed every six months.

#### Assessment of physical fitness

Physical fitness assessments include the 550 m walk/run [43], and 5 days of ActiGraph wGT3X-BT (Actigraph, Pensacola, Florida, USA) accelerometer wear will be requested (3 weekdays and 2 weekend days), giving an estimated reliability of 0.80 in children and 0.70 in adolescents [44]. These levels of reliability have been questioned in more recent studies [45], however longer wear time is not practical due to resource. Epoch time will be set to 60 s and cut-off time 60 min.

#### Metabolic markers

Venous blood sampling will be undertaken to assess for metabolic status (Table 2). These biomarkers identify the biochemical comorbidities associated with obesity [46, 47]. Incentivisation for these samples will be provided.

#### Comorbidities

Weight-related comorbidities will be screened for and referrals will be made where appropriate.

### Study arms

Every child entering Whanau Pakari will be discussed at the multi-disciplinary team meeting, allowing a full clinical review and referrals for further investigation where appropriate, with additional discussion of dietary intake, physical activity, and psychology issues. The assessments and multi-disciplinary meetings will be repeated for each child/adolescent every six months, irrespective of which group they are randomised to.

#### Intervention

The intervention programme will be a 12-month multi-disciplinary programme with weekly group sessions. It will be administered by a physical activity coordinator, community dietitian, and psychologist: all staff members within the Whanau Pakari team. It will involve:

1. Home visits with the dietitian and physical activity coordinator in the initial phase
2. Weekly contact in activity sessions of either physical activity OR
3. Psychology sessions (covering topics such as bullying, self-esteem, parenting, making lifestyle changes), and dietitian sessions (covering topics such as portion size, virtual supermarket tours, healthy food on a budget, and vegetable gardens) (Table 3).

**Table 3:** Support provided to each group of trial participants enrolled in Whanau Pakari

	Control (Current “standard care”)	Intervention
6, 12, 18, 24 month assessments with nutrition advice and feedback (blood tests at baseline, 12, 24 months)	✓	✓
Home visit within 1^st^ month from physical activity coordinator and dietitian		✓
Physical activity coordinator/Dietitian review of progress at 6 months (seen at group)		✓
Questionnaire review (team), multi-disciplinary team meeting – review and action of alerts	✓	✓
+/− Keyworker		✓
Weekly activity and education sessions for 12 months		✓
Total home visits over 2 years	5	6

The participants will be engaged in the programme one hour per week for four school terms (equating to a total of 40 sessions). The same programme will be delivered to all participants, however these will be tailored to meet the cultural requirements of participants, for example, sessions that include traditional Maori games, recipe makeover of dishes from different cultures, and honouring of particular dietary requirements in cooking sessions. A committed family member will be required to attend to support and learn alongside the child/teenager, as the programme is family-focussed. No incentivisation will be provided for attending sessions. A commitment contract will be signed at entry to the programme, outlining expectations of attendance at a minimum of 70 % of sessions to gain the most out of the programme. After 12 months, families will be linked into local sport centres/aquatic centres/gym facilities.

#### Non-intervention

The non-intervention (control) group will receive the same home-visit model and assessments as the intervention group, but will not undertake any of the intervention sessions (Table 3). The families will receive feedback from the assessments with dietary and physical activity guidelines. This model was chosen for community acceptability and is very similar to what is currently considered ‘standard care’. This group are offered the intervention after 24 months. All participants declining intervention and those who have completed intervention and 12 month follow-up will be discharged back to primary care, with a guideline sheet of what to monitor, and assess with regard to weight-related comorbidities. Any participants withdrawing from the trial before the end of the 24 months will be incentivised to participate in a modified assessment to allow measurement of their progress, thus maximising completeness of data for the intention to treat analysis.

### Randomisation

At baseline, there will be 2 assessments of RFC (given it is not known if a quantitative tool will be successful): the clinician’s subjective measure (ranked pre-contemplation, contemplation, preparation/action), and a specifically devised questionnaire using a 5-point Likert scale that is completed by the child (if > 11 years of age) and a second one for the parent. The RFC questionnaire was based on the original readiness to change questionnaire [15], focussing on beliefs and behaviour around three factors: weight, eating habits and activity levels. If the RFC ranking is scored in the contemplative or further along on either scale, then participants will be offered entry into the trial. We purposely set the bar low (i.e. below the preparation/action level) to assess whether degree of RFC predicts outcomes within those contemplative or above.

Patients will be assessed, consented, and entered into the trial prior to randomisation. Randomisation by minimisation (using age and ethnicity) will be conducted using the Minim randomisation computer programme which maintains approximate balance in the 2 arms for age and ethnicity but incorporates a random element so it cannot be predicted which study arm the subject will be allocated to.

### Primary outcome

The primary outcome measure is the change in BMI SDS in the intervention group compared with the control group recorded at 6, 12, 18 and 24 months post enrolment.

### Secondary outcomes

Secondary outcomes include changes in health-related quality of life; dietary knowledge and behaviour; physical activity (specifically moderate to very vigorous physical activity), sedentary behaviour, knowledge of benefits of physical activity, cardiovascular and metabolic profile (blood pressure, resting heart rate, waist circumference and WHtR) all at 6, 12, 18, and 24 months, and glycaemic control (fasting glucose and HbA1c), fasting insulin and lipids at baseline, 12 and 24 months.

### Other aims and outcomes

This study also aims to investigate whether those assessed as ready for change (i.e. preparation/action) experience a greater reduction in BMI SDS compared with those less ready for change (i.e. contemplation). If the intervention is found to be effective, a cost-effectiveness analysis will be undertaken, taking into account multiple outcome parameters.

### Data collection

The Healthy Lifestyles Coordinator will collect all data (apart from physical activity assessments) in the home assessments.

All data from assessments will be entered into a specific purpose-built database, which will be reviewed monthly for data validity and completeness. This database includes alerts for data outside acceptable medical parameters, for example, elevated blood pressure percentiles, for discussion at multi-disciplinary meetings.

Progress will be captured on the multi-disciplinary team meeting database page, so the team can review the results of each 6-month assessment for each participant, and make further recommendations or referrals.

### Statistical considerations

#### Sample size

With 107 participants per group (120 to account for a 10 % dropout rate) there is 80 % power to detect a difference in change in BMI of 0.5 SDS at the 5 % level of significance, with a standard deviation of 1.3 [48]. Of note, only 15/54 lifestyle studies included in a systematic review at the time of study design reported power calculations [11]. Subsequent meta-analysis of interventions has shown that even a change of −0.1 BMI SDS can lead to improvements in cardiovascular and metabolic outcomes [12]. It is hoped that even demonstrating smaller differences as this more recent literature has shown will still be important at a population level.

#### Data analyses

Statistical analyses will be performed using SAS version 9.3 (SAS Institute Inc. Cary NC). A general linear mixed model will be used to assess change from baseline measured at 6, 12, 18 and 24 months after initiating the intervention adjusting for age, gender, socioeconomic status, and ethnicity and degree of readiness for change. Means and standard deviations of changes from baseline in outcomes of interest, for both the raw and modelled data, will be presented.

#### Baseline characteristics

Baseline characteristics will be summarised using descriptive statistics. Continuous variables will be described as numbers of observed and missing values, mean, standard deviation, median, minimum and maximum. Categorical variables will be described as frequencies and percentages.

#### Treatment effects

Analyses will be performed on the intended to treat population. Reporting will adhere to the CONSORT guidelines for reporting parallel group randomised trials [13].

## Discussion

Whanau Pakari is expected to provide important new knowledge to the area of child obesity. This will be achieved with its focus on an indigenous group at increased risk for obesity and its resulting comorbidities, and through its engagement with the community to increase acceptability of the programme. It is likely to inform these areas in a robust manner if high participation is achieved and maintained.

This randomised controlled trial is unique in three key ways. First, it is assessing a mainstream multi-disciplinary clinical service that has evolved to specifically ensure accessibility and appropriateness for Indigenous people. Whanau Pakari is a community “real life” intervention programme resulting out of a clinical need, and has the potential to answer critical questions in relation to delivery of interventions in this area. Secondly, Whanau Pakari utilises a home-visit model which replaces hospital medical assessments, therefore “de-medicalising” obesity assessments. The family-based home model is likely to appeal to many ethnic groups who resist the hospital-based mainstream clinical models currently operating in most areas. Thirdly, Whanau Pakari will only include those considered potentially ready for change, and will investigate whether the level of “readiness” to make lifestyle changes predicts improved outcomes in intervention programmes. If the RFC measures provide a reliable and valid measure of outcome success, then the development of paired interventions around motivation for change for those in earlier stages of change followed by direct interventions for those in later stages could result in less programme dropout as well as being a more efficient and cost effective utilisation of limited resource.

A limitation of this study is the use of self-report for Tanner pubertal stage. Previous literature has demonstrated that obese girls tend to overestimate breast size and obese males stage of pubic hair development more than their non-obese counterparts [49]. However, we did not believe it was appropriate to undertake pubertal examination within the home setting, and it was determined this would be a more appropriate cut-off for pre-pubertal and pubertal status than an arbitrary age for males and females.

Weight-related comorbidities are a particular concern in child obesity, given the long-lasting effects of these conditions [50]. Past meta-analysis highlighted that a BMI SDS reduction of −0.1 led to significant improvements in multiple cardiovascular and metabolic outcomes over time [12]. BMI, in conjunction with waist circumference, WHtR, fasting lipids, glucose and insulin, and blood pressure (all being measured at intervals over an extended period of follow-up) will provide a comprehensive assessment of cardiovascular and metabolic outcomes long-term.

Whilst there has been a shifting focus toward early obesity intervention targeting critical periods of human development, there is still a need for an effective intervention programme to provide assistance for those children and adolescents who are already obese. This research aligns with the Ministry of Health’s clinical guidelines for weight management in children and young people [7], and addresses barriers to accessing services and programmes identified by the Office of the Auditor General’s performance audit into child obesity [51].

In summary, this trial will determine if this unique multi-disciplinary intervention will result in improved health outcomes, especially among Maori. It will also investigate whether there is an indication that being at the preparation/action stages of RFC compared to those in the contemplative stage results in improved success in intervention programmes. As translational research, it will inform the New Zealand Ministry of Health regarding ways to combat child and adolescent obesity. It is hoped this study will lead to prevention of the adult associated comorbidities of child obesity into later life for some individuals, thereby reducing morbidity, particularly for those most vulnerable in our population.

## Abbreviations

ANZCTR: Australian New Zealand Clinical Trials Registry
OECD: Organisation for Economic Cooperation and Development
CONSORT: Consolidated Standards of Reporting Trials
RFC: Readiness for change
BMI: Body mass index
HbA1c: glycated Haemoglobin
SDS: standard deviation score
WHtR: Waist height ratio
Peds QL: Pediatric Quality of Life
CBCL: Child Behaviour Checklist
C-PAQ: Children’s Physical Activity Questionnaire
CDQ: Children’s Dietary Questionnaire

## References

[1] Lobstein T, Baur L, Uauy R, IASO International Obesity TaskForce (2004). Obesity in children and young people: a crisis in public health. Obes Rev.

[2] Han JC, Lawlor DA, Kimm SY (2010). Childhood obesity. Lancet.

[3] Baur LA, Hazelton B, Shrewsbury VA (2011). Assessment and management of obesity in childhood and adolescence. Nat Rev Gastroenterol Hepatol.

[4] OECD. “Overweight and obesity”, in OECD Factbook 2013: Economic, Environmental and Social Statistics. http://dx.doi.org/10.1787/factbook-2013-100-en.

[5] Ministry of Health (2014). Annual update of key results 2013/2014: New Zealand health survey.

[6] Anderson YC, Taylor GM, Grant CC, Fulton RB, Hofman PL (2015). The Green Prescription Active Families programme in Taranaki, New Zealand 2007–2009: did it reach children in need?. J Prim Health Care.

[7] Ministry of Health and Clinical Trials Research Unit, New Zealand. Clinical guidelines for weight management in New Zealand children and young people. 2009. http://www.health.govt.nz/publication/clinical-guidelines-weight-management-new-zealand-children-and-young-people.

[8] Anderson YC, Cave TL, Cunningham VJ, Pereira NM, Woolerton DM, Grant CC (2015). Effectiveness of current interventions in obese New Zealand children and adolescents. NZMJ.

[9] Cretikos MA, Valenti L, Britt HC, Baur LA (2008). General practice management of overweight and obesity in children and adolescents in Australia. Med Care.

[10] Wake M, Campbell MW, Turner M, Price A, Sabin MA, Davis E (2013). How training affects Australian paediatricians’ management of obesity. Arch Dis Child.

[11] Oude Luttikhuis H, Baur L, Jansen H, Shrewsbury VA, O'Malley C, Stolk R, et al. Interventions for treating obesity in children. Cochrane Database Syst Rev. 2009; Issue 1. Art. No.: CD001872. doi:10.1002/14651858.CD001872.pub2. 10.1002/14651858.CD001872.pub2 19160202

[12] Ho M, Garnett SP, Baur L, Burrows T, Stewart L, Neve M (2012). Effectiveness of lifestyle interventions in child obesity: systematic review with meta-analysis. Pediatrics.

[13] Moher D, Hopewell S, Schulz KF, Montori V, Gotzsche PC, Devereaux PJ (2010). CONSORT 2010 explanation and elaboration: updated guidelines for reporting parallel group randomised trials. BMJ.

[14] Prochaska JO, DiClemente CC (1982). Transtheoretical therapy: toward a more integrative model of change. Psychother Theor Res Prac Fal.

[15] Rollnick S, Heather N, Gold R, Hall W (1992). Development of a short “readiness to change” questionnaire for use in brief, opportunistic interventions among excessive drinkers. Br J Addict.

[16] DiClemente CC, Prochaska JO (1982). Self-change and therapy change of smoking behavior: a comparison of processes of change in cessation and maintenance. Addict Behav.

[17] Hoke M, Timmerman G (2011). Transtheoretical model: potential usefulness with overweight rural Mexican American women. Hisp Health Care Int.

[18] Rhee KE, De Lago CW, Arscott-Mills T, Mehta SD (2005). Factors associated with parental readiness to make changes for overweight children. Pediatrics.

[19] Statistics New Zealand. Census 2013: ethnic group by age group and sex. http://nzdotstat.stats.govt.nz/wbos/Index.aspx?DataSetCode=TABLECODE8021#.

[20] Cole TJ (2002). A chart to link child centiles of body mass index, weight and height. Eur J Clin Nutr.

[21] Ministry of Health, New Zealand. Child health: fact sheet 6, plotting and assessing infants and toddlers up to age five years. http://www.health.govt.nz/system/files/documents/pages/factsheet-6-growth-charts-well-child.pdf.

[22] Ministry of Health, New Zealand. He Korowai Oranga: Maori health strategy 2014. http://www.health.govt.nz/publication/guide-he-korowai-oranga-maori-health-strategy.

[23] Ministry of Health, New Zealand. The Whanau Ora tool. http://www.publichealthworkforce.org.nz/data/media/documents/Maori%20PHWD/Final%20Whanau%20Ora%20A4-4.pdf.

[24] Glover M, Kira A, Johnston V, Walker N, Thomas D, Chang A (2015). A systematic review of barriers and facilitators to participation in randomized controlled trials by Indigenous people from New Zealand, Australia, Canada and the United States. Glob Health Promot.

[25] Freeman JV, Cole TJ, Chinn S, Jones PR, White EM, Preece MA (1995). Cross sectional stature and weight reference curves for the UK, 1990. Arch Dis Child.

[26] Australasian Paediatric Endocrine Group. Australian and New Zealand growth charts. http://www.apeg.org.au/ClinicalResourcesLinks/GrowthGrowthCharts/tabid/101/Default.aspx.

[27] National Center for Health Statistics. Clinical growth charts: Centers for Disease Control and Prevention. www.cdc.gov/nchs/about/major/nhanes/growthcharts/datafiles.htm.

[28] National High Blood Pressure Education Program Working Group on High Blood Pressure in Children and Adolescents (2004). The fourth report on the diagnosis, evaluation, and treatment of high blood pressure in children and adolescents. Pediatrics.

[29] Shypailo RJ, Ellis KJ. Age-based pediatric blood pressure reference charts. https://www.bcm.edu/bodycomplab/Flashapps/BPVAgeChartpage.html.

[30] St George IM, Reid JJ, Grimmond BB, Morton R (1982). PEFR in Dunedin children aged 5–15 years. N Z Med J.

[31] Tanner J (1962). Growth at adolescence; with a general consideration of the effects of hereditary and environmental factors upon growth and maturation from birth to maturity.

[32] University of Otago - School of Medicine and Health Science, New Zealand. NZ Deprivation Index 2006. https://koordinates.com/layer/1066-nz-deprivation-index-2006/.

[33] Varni JW, Seid M, Rode CA (1999). The PedsQL: measurement model for the pediatric quality of life inventory. Med Care.

[34] Varni JW, Seid M, Kurtin PS (2001). PedsQL 4.0: reliability and validity of the Pediatric Quality of Life Inventory version 4.0 generic core scales in healthy and patient populations. Med Care.

[35] Varni JW, Burwinkle TM, Seid M, Skarr D (2003). The PedsQL 4.0 as a pediatric population health measure: feasibility, reliability, and validity. Ambul Pediatr.

[36] Varni JW, Seid M, Knight TS, Uzark K, Szer IS (2002). The PedsQL™ 4.0 generic core scales: sensitivity, responsiveness, and impact on clinical decision-making. J Behav Med.

[37] Chan KS, Mangione-Smith R, Burwinkle TM, Rosen M, Varni JW (2005). The PedsQL: reliability and validity of the short-form generic core scales and asthma module. Med Care.

[38] Varni JW, Limbers CA (2009). The PedsQL™ 4.0 generic core scales young adult version: feasibility, reliability and validity in a university student population. J Health Psychol.

[39] Achenbach TM, Ruffle TM (2000). The Child Behavior Checklist and related forms for assessing behavioral/emotional problems and competencies. Pediatr Rev.

[40] Corder K, van Sluijs EM, Wright A, Whincup P, Wareham NJ, Ekelund U (2009). Is it possible to assess free-living physical activity and energy expenditure in young people by self-report?. Am J Clin Nutr.

[41] Magarey A, Golley R, Spurrier N, Goodwin E, Ong F (2009). Reliability and validity of the Children's Dietary Questionnaire: a new tool to measure children's dietary patterns. Int J Pediatr Obes.

[42] Clinical Trials Research Unit and Synovate. A national survey of children and young people's physical activity and dietary behaviours in New Zealand: 2008/09 - key findings. http://www.health.govt.nz/publication/national-survey-children-and-young-peoples-physical-activity-and-dietary-behaviours-new-zealand-2008.

[43] Hamlin MJ, Fraser M, Lizamore CA, Draper N, Shearman JP, Kimber NE (2014). Measurement of cardiorespiratory fitness in children from two commonly used field tests after accounting for body fatness and maturity. J Hum Kinet.

[44] Trost SG, Pate RR, Freedson PS, Sallis JF, Taylor WC (2000). Using objective physical activity measures with youth: How many days of monitoring are needed?. Med Sci Sports Exerc.

[45] Ojiambo R, Cuthill R, Budd H, Konstabel K, Casajús JA, González-Agüero A (2011). Impact of methodological decisions on accelerometer outcome variables in young children. Int J Obes.

[46] Lakshman R, Elks CE, Ong KK (2012). Childhood obesity. Circulation.

[47] Barness LA, Opitz JM, Gilbert-Barness E (2007). Obesity: genetic, molecular, and environmental aspects. Am J Med Genet A.

[48] Webber L, Hill C, Saxton J, Van Jaarsveld CH, Wardle J (2009). Eating behaviour and weight in children. Int J Obes (Lond).

[49] Bonat S, Pathomvanich A, Keil MF, Field AE, Yanovski JA (2002). Self-assessment of pubertal stage in overweight children. Pediatrics.

[50] World Health Organization. Interim Report of the Commission on Ending Childhood Obesity. 2015.

[51] Controller and Auditor General (2013). Evolving approach to combating child obesity.

[52] World Health Organisation. Waist circumference and waist-hip ratio: report of a WHO expert consultation. http://www.who.int/nutrition/publications/obesity/WHO_report_waistcircumference_and_waisthip_ratio/en/.

